# Do ultrathin strut bare-metal stents with passive coating improve efficacy in large coronary arteries? Insights from the randomized, multicenter BASKET-PROVE trials

**DOI:** 10.1186/s12872-019-1199-8

**Published:** 2019-10-16

**Authors:** Kim Wadt Hansen, Raban Jeger, Rikke Sørensen, Christoph Kaiser, Matthias Pfisterer, Tor Biering-Sørensen, Louise Hougesen Bjerking, Søren Galatius

**Affiliations:** 1 Department of Cardiology, University Hospital Bispebjerg and Frederiksberg, DK-2400 Copenhagen, NV Denmark; 2 Department of Cardiology, University Hospital Basel, Basel, Switzerland; 3 Department of Cardiology, Rigshospitalet, Copenhagen, Denmark; 4 Department of Cardiology, University Hospital Herlev and Gentofte, Copenhagen, Denmark

**Keywords:** Coronary artery disease, Bare-metal stents, Treatment outcome

## Abstract

**Background:**

The new generation thinner-strut silicon carbide (SiC) coated cobalt chromium (CoCr) bare-metal stents (BMS) are designed to accelerate rapid endothelialisation and reduce thrombogenicity when implanted in coronary arteries. However, smaller studies suggest higher rates of symptomatic restenosis in patients receiving the newer generation BMS.

We investigated the efficacy of a newer generation ultrathin strut silicon-carbide coated cobalt-chromium (CoCr) BMS (SCC-BMS) as compared to an older thin-strut uncoated CoCr BMS (UC-BMS) in patients presenting with coronary artery disease requiring stenting of large vessels (≥3.0 mm).

**Methods:**

All patients randomized to SCC- (*n* = 761) or UC-BMS (*n* = 765) in the two BASKET-PROVE trials were included. Design, patients, interventions and follow-up were similar between trials except differing regimens of dual antiplatelet therapy. The primary endpoint was clinically driven target-vessel revascularization within 24 months. Safety endpoints of cardiac death, non-fatal myocardial infarction (MI), and definite/probable stent thrombosis (ST) were also assessed. We used inverse probability weighted proportional hazards Cox regressions adjusting for known confounders.

**Results:**

Demographics, clinical presentation, and risk factors were comparable between the groups, but patients receiving SCC-BMS underwent less complex procedures. The risk for clinically driven TVR was increased om the SCC-BMS group compared to the UC-BMS group (cumulative incidence, 10.6% vs. 8.4%; adjusted relative hazard [HR], 1.49 [95% CI, 1.05–2.10]). No differences in safety endpoints were detected, cardiac death (1.6% vs. 2.8%; HR, 0.62 [CI, 0.30–1.27]), non-fatal MI (3.2% vs. 2.5%; HR, 1.56 [CI, 0.83–2.91]), and definite/probable ST (0.8% vs. 1.1%; HR, 1.17 [CI, 0.39–3.50]). Differences in strut thickness between the two stents did not explain the association between stent type and clinically driven TVR.

**Conclusions:**

In patients requiring stenting of large coronary arteries, use of the newer generation SCC-BMS was associated with a higher risk of clinically driven repeat revascularization compared to the UC-BMS with no signs of an offsetting safety benefit.

## Background

Drug-eluting stents (DES) constitute the standard of care in revascularization in the context of modern dual antiplatelet therapy [1–3]. The new generation ultrathin-strut cobalt-chromium (CoCr) bare-metal stents (BMS) with a passive silicon carbide coating were designed to rival modern DES through reduced rates of in-stent restenosis [4]. Although initial findings from clinical registries were promising with rates of target-lesion revascularization (TVR) below 5% at 6 months [5, 6] subsequent observational analyses using an established uncoated CoCr BMS as comparator demonstrated higher rates of TVR at 12 and 18 months [7, 8]. The randomized, multicenter BAsel Stent Kosten-Effektivitäts Trial-PROspective Validation Examination (BASKET-PROVE) and BASKET-PROVE II trials provide a unique opportunity for studying this relation in patients with large coronary arteries, who a priori have a lower risk of symptomatic restenosis, at 24 months follow-up.

We investigated the efficacy of a new generation ultrathin strut silicon-carbide coated CoCr BMS (SCC-BMS) as compared to an older thin strut uncoated CoCr BMS (UC-BMS) in patients with coronary artery disease requiring stenting of large vessels (≥3.0 mm).

## Methods

### Data sources, patients and design

#### Data sources

We conducted a post-hoc analysis of the BASKET-PROVE and BASKET-PROVE II trials. A detailed description of study design, methods and primary findings has been provided elsewhere [9–12]. In brief, both trials included patients presenting with chronic or acute coronary artery disease requiring angioplasty and stenting with stents ≥3.0 mm in diameter. Exclusion criteria were: cardiogenic shock, in-stent restenosis or thrombosis, unprotected left main coronary disease or bypass-graft disease, planned surgery within 12 months, need for oral anticoagulation, increased bleeding risk, known intolerance to or suspected noncompliance with long-term antiplatelet therapy, or circumstances that would have made follow-up impossible. Study procedures including angioplasty and stenting were performed using standard techniques left at the discretion of each interventional cardiologist. Lifelong aspirin at a dose of 75 to 100 mg was prescribed for all patients. Prescription of other concomitant medication, i.e. statin, followed current guidelines. Angiography and revascularization during follow-up were performed only if clinically indicated.

#### Patients, stent designs and P_2_Y_12_ inhibitors

The BASKET-PROVE trial enrolled 2314 patients between March 5, 2007 and May 15, 2008 at 11 participating European centers; a total of 765 patients were allocated to receive a thin-strut uncoated CoCr bare-metal stent (UC-BMS; [Vision, Abbott Vascular]). This stent was uncoated with a multilink architecture and strut thickness (81 μm) was consistent regardless of stent diameter. All patients were prescribed clopidogrel at a daily dose of 75 mg for 12 months after a loading dose of 600 mg, regardless of stent type. The BASKET-PROVE II trial enrolled 2291 patients between April 1, 2010 and May 21, 2012 at eight participating European centers; 761 of which were allocated to receive a newer generation ultrathin strut silicon-carbide coated CoCr bare-metal stent (SCC-BMS; [PRO-Kinetic, Biotronik]). By design this stent had a double-helical structure and a strut thickness which depended on stent diameter; i.e. the relation between stent diameter and strut thickness was 2.5/3.0 mm ~ 60 μm, 3.5/4.0 mm ~ 80 μm, and 4.5 mm ~ 120 μm, respectively. Patients received a loading dose of 60 mg prasugrel with a maintenance dose of 10 mg daily, risk-adjusted to 5 mg in patients more than 75 years of age or a body weight of less than 60 kg. Prasugrel was prescribed for 12 months except in patients with stable CAD receiving a bare-metal stent where duration was 4 weeks. Patients with a prior history of stroke or transient ischaemic attack were excluded from the BASKET-PROVE II trial due to the use of prasugrel as antiplatelet therapy, which was not the case in the BASKET-PROVE trial.

#### Design

The present analysis compared patients randomized to a SCC-BMS (plus prasugrel) with those randomized to a UC-BMS (plus clopidogrel) in terms of repeat revascularization and ischaemic cardiac events during 24 months of follow-up in patients with diseased large coronary vessels. The study protocols were approved by the local ethics committee at each participating site and all patients had given their written informed consent. The authors have full access to the data and take responsibility for its integrity. All authors have read and approved the manuscript.

### Endpoint definitions

The primary endpoint was any clinically driven target-vessel revascularization at 24 months. The main secondary endpoint was a composite of cardiac death, non-fatal myocardial infarction, or definite/probable stent thrombosis; henceforth referred as ischaemic cardiac events. Additional secondary endpoints included major adverse cardiac events (composite of cardiac death, non-fatal MI and non-MI-related TVR), individual components of the main secondary endpoint, and all-cause death. Target vessel revascularization included any revascularization of any vessel treated by PCI and stenting at baseline. Any death without a clear extracardiac cause was classified as a cardiac death. Myocardial infarction was defined as a clinical event with typical electrocardiographic or enzymatic changes, and stent thrombosis was defined according to the Academic Research Consortium (ARC) criteria [13].

### Statistical analysis

Patient- and procedure-related characteristics are reported as counts (percentages) or mean (sd). Absolute time-to-event measures were calculated using cumulative incidence curves accounting for death from all causes as a compering risk [14].

We calculated adjusted relative hazard estimates using proportional hazards Cox regression analyses applying inverse probability weighting to adjust for any confounding due to differences in baseline and procedural characteristics between the treatment groups [15]. A propensity score model was computed by fitting a non-parsimonious logistic regression with allocation to the SCC-BMS group being the dependent variable, and patient- and procedure-related characteristics measured at baseline being the independent variables. An inverse probability weight was calculated for each patient using the estimated propensity scores. This approach involved weighting each patient who received a SCC-BMS by the inverse of the probability that he or she would be selected for the SCC-BMS group; and weighting each patient who did not receive a SCC-BMS by the inverse probability that he or she would not be selected for the SCC-BMS group. We used stabilized inverse probability weights and assessed the performance of the weighting by comparing the distribution of covariates between treatment groups [16].

Patient characteristics included age and dichotomized variables (yes vs. no): sex (male vs. female), clinical presentation (stable angina vs. acute coronary syndrome), hypertension, hyperlipidemia, diabetes mellitus, active smoking, prior myocardial infarction, prior percutaneous coronary intervention, prior coronary artery bypass-grafting surgery, heart failure, chronic obstructive pulmonary disease, peripheral arterial occlusive disease, stroke or transient ischaemic attack, and cancer. Procedural characteristics included no. of treated segments per patient, no. of stents per patients, and total stented length (mm) along with dichotomized variables: bifurcation lesion, chronic total occlusion, multivessel disease, additional use of stent < 3.0 mm in diameter, diseased left main coronary artery (LM), diseased left anterior descending artery (LAD), diseased left circumflex artery (LCx), diseased right coronary artery (RCA), use of any GPIIb/IIIa blocker, and staged procedure. Data on procedural characteristics were missing for one patient in the UC-BMS group. This was addressed by imputing missing values with the UC-BMS group-specific mean for each variable.

Model assumptions were valid unless otherwise stated. The assumption of constant hazards was assessed through log-log survival curves and by testing Schoenfeld’s residuals for time-dependency. All hypothesis tests had a two-sided significance level of 0.05. Analyses were performed on the intention-to-treat population using statistical software R, version 3.2.2 [17].

## Results

### Patients

All 1526 patients randomly allocated to the UC-BMS group (*n* = 765) or the SCC-BMS group (*n* = 761) were included in the analysis. The median follow-up in surviving patients was 733 days (IQR: 706–758 days). Overall, the mean age was 63.8 years, 75.8% were males, and 61.7% presented with an acute coronary syndrome. Baseline characteristics are shown in Table 1. Patients in the SCC-BMS group were slightly younger and a history of diabetes mellitus, active smoking, and prior PCI tended to be more prevalent, while prior MI and a clinical presentation with STEMI were less frequent compared to those in the UC-BMS group.

**Table 1:** Patient characteristics at baseline

	Silicon carbide-coated BMS	Uncoated cobalt-chromium BMS	*P*-value^a^
No. patients	761	765	
Age, y (median, [IQR])	63 [55–71]	64 [57–72]	0.11
Male sex	570 (74.9)	586 (76.6)	0.48
Cardiac risk factors			
Arterial hypertension	510 (67.0)	485 (63.4)	0.15
Hyperlipidemia	471 (61.9)	495 (64.7)	0.28
Diabetes mellitus	141 (18.5)	108 (14.1)	0.024
Active smoker	502 (66.0)	464 (60.7)	0.036
Prior MI	75 (9.9)	100 (13.1)	0.059
Prior PCI	115 (15.1)	88 (11.5)	0.046
Prior CABG	14 (1.8)	20 (2.6)	0.39
Comorbidity			
Heart failure	45 (5.9)	53 (6.9)	0.48
Prior stroke/TIA	8 (1.1)	31 (4.1)	<0.001
PAOD	34 (4.5)	30 (3.9)	0.69
COPD	55 (7.2)	48 (6.3)	0.52
Cancer	28 (3.7)	37 (4.8)	0.32
Clinical presentation			
Stable angina	300 (39.4)	285 (37.3)	0.41
NSTE-ACS	253 (33.2)	246 (32.2)	0.69
STEMI	208 (27.3)	234 (30.6)	0.18

No. are counts (%), unless otherwise indicated

^a^Mann-Whitney U test if continuous variable; chi-squared test if categorical variable

*BMS* bare-metal stent, *CABG* coronary artery bypass-grafting surgery, *COPD* chronic obstructive pulmonary disease, *MI* myocardial infarction, *NSTE-ACS* non-ST-segment elevation acute coronary syndrome, *PAOD* peripheral arterial occlusive disease, *PCI* percutaneous coronary intervention, *STEMI* ST-segment elevation myocardial infarction

### Procedures

Procedure-related characteristics are listed in Table 2. Patients in the SCC-BMS group presented with less bifurcational lesions and were less likely to receive additional stents < 3.0 mm in diameter or GPIIb/IIIa inbibitors. Number of treated segments, number of stents per patient, total stented length per patient and consequently stented length per lesion were all significantly lower in the SCC-BMS versus UC-BMS group. Prescription of aspirin, P2Y12 receptor inhibitors, statins, and anticoagulation therapy at discharge was comparable between the treatment groups (Table 3).

**Table 2:** Procedure-related characteristics

	Silicon carbide-coated BMS	Uncoated cobalt-chromium BMS	*P*-value
Patients, no.	761	765	
Treated segments, no.	962	1117	
Treated vessels			
Left main artery (protected)	3 (0.4)	9 (1.2)	0.15
Left anterior descending artery	491 (64.5)	496 (64.8)	0.94
Left circumflex artery	252 (33.1)	279 (36.5)	0.19
Right coronary artery	394 (51.8)	414 (54.1)	0.39
Complexity of CAD^a^			
Multivessel disease	299 (39.3)	327 (42.7)	0.19
Bifurcational lesion	45 (5.9)	68 (8.9)	0.033
Chronic total occlusion	26 (3.4)	39 (5.1)	0.13
Stent <3.0 mm	20 (2.6)	38 (5.0)	0.024
GPIIb/IIIa blocker use	93 (12.2)	168 (22.0)	<0.001
Procedural characteristics^a^, ^b^			
Segments per patient, no.	1.3 ± 0.5	1.5 ± 0.8	<0.001
Stents per patient, no.	1.5 ± 0.8	1.7 ± 1.1	<0.001
Total stent length, mm	25.1 ± 15.7	31.2 ± 22.5	<0.001
Stent length per lesion, mm	19.7 ± 7.9	21.1 ± 9.6	0.017
Maximum deployment pressure, mmHg	14.7 ± 3.4	15.1 ± 3.3	0.003
Staged procedure	45 (5.9)	33 (4.3)	0.20
Lesions with angiographic success, no.	917 (95.3)	1080 (96.7)	0.14

No. are counts (%), unless otherwise indicated. *BMS* bare-metal stent, *CAD* coronary artery disease

^a^Missing for 1 patient in the UC-BMS group

^b^Plus-minus values are mean ± SD

**Table 3:** Discharge medication

	Silicon carbide-coated BMS	Uncoated cobalt-chromium BMS	*P*-value
Patients, no.	761	765	
Aspirin	757 (99.5)	764 (99.9)	0.37
P2Y12-inhibitor^a^	757 (99.5)	764 (99.9)	0.37
Anticoagulation therapy^b^	29 (3.8)	34 (4.5)	0.63
Statin	715 (94.3)	706 (93.0)	0.35
Antithrombotic strategies			
Dual antiplatelet therapy	754 (99.1)	763 (99.7)	0.18
Triple therapy^a^	27 (3.6)	34 (4.5)	0.45

No. are counts (%), unless otherwise indicated. *BMS* bare-metal stent

^a^Clopidogrel (75 mg) or Prasugrel (5 or 10 mg)

^b^Missing = 40

### Outcomes

**Fig. 1:**
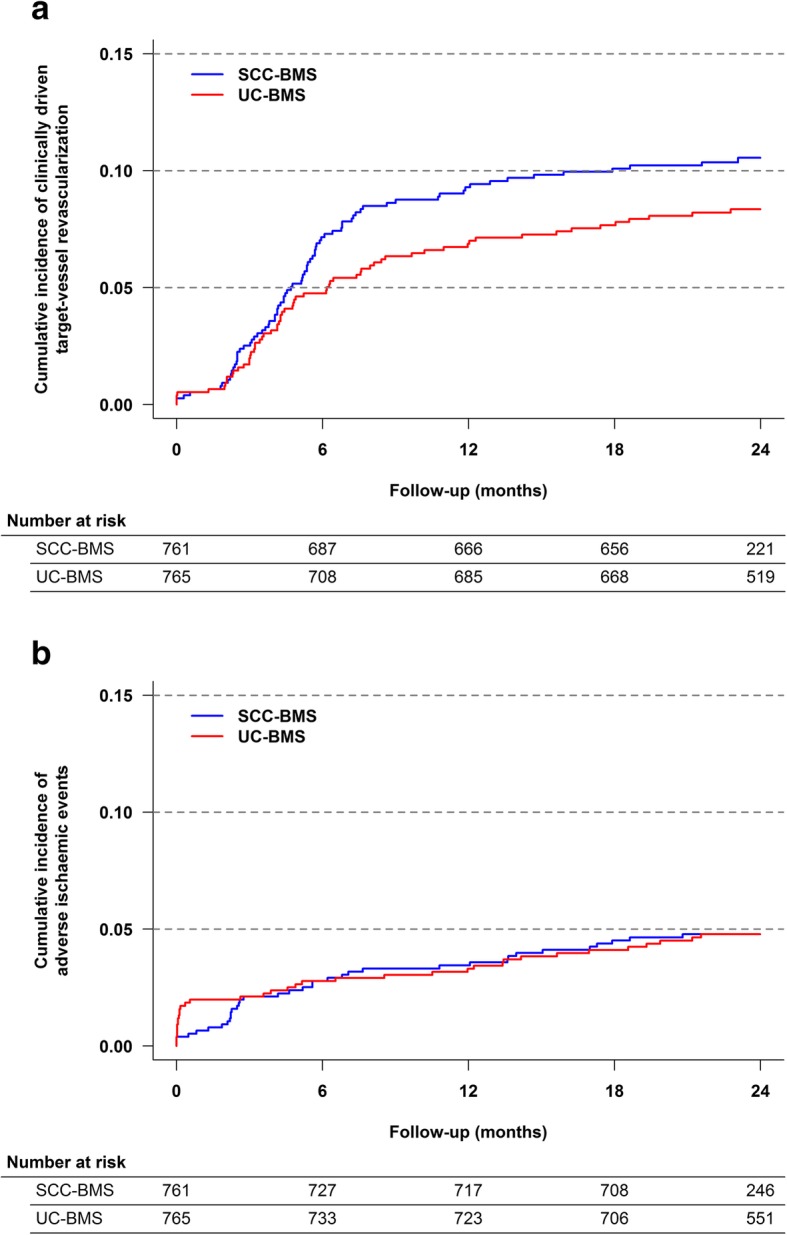
Cumulative incidence curves for clinically driven target-vessel revascularization and ischaemic cardiac events within 24 months

All patient and procedure-related characteristics were well balanced after applying the stabilized inverse probability weights (Additional file 1: Figure S1). The unadjusted cumulative incidence curves for clinically driven TVR and ischaemic cardiac events within 24 months are shown in Fig. 1. Notably, the curves for clinically driven TVR diverge between 5- and 8-months following stent implantation. At 24 months receipt of a SCC-BMS was associated with a higher risk for clinically driven target-vessel revascularization compared to a UC-BMS (cumulative incidence, 10.5% vs. 8.4%; adjusted hazard ratio [HR], 1.48 [CI 95%, 1.05–2.09]; *p* = 0.024). Overall, no differences in ischaemic cardiac events were observed (cumulative incidence, 4.8% vs. 4.7%; adjusted HR, 1.21 [CI 95%, 0.75–1.95]; *p* = 0.43). No significant differences were observed for cardiac death (cumulative incidence, 1.6% vs. 2.8%; adjusted HR, 0.66 [CI 95%, 0.32–1.36]; *p* = 0.26) and non-fatal MI (cumulative incidence, 3.2% vs. 2.6%; adjusted HR, 1.62 [CI 95%, 0.87–3.01]; *p* = 0.13) in the SCC-BMS group compared to the UC-BMS group. No discernable differences were observed for definite/probable ST (cumulative incidence, 0.8% vs. 1.1%; adjusted HR, 1.21 [CI 95%, 0.41–3.56]; *p* = 0.73) and all-cause death (cumulative incidence, 3.4% vs. 4.3%; adjusted HR, 0.79 [CI 95%, 0.46–1.36]; *p* = 0.40). Outcomes are summarized in Table 4 and additional unadjusted cumulative incidence curves in Additional file 1: Figures S2 and S3.

**Table 4:** Cumulative incidences and relative hazards for individual and composite endpoints at 24 months

	Events, no. (cumulative incidence per 100 patients at risk)		Hazard ratio (95% CI)		
	SCC-BMS	UC-BMS	Unadjusted	Adjusted^a^	*P*-value^a^
Individual endpoints					
Target-vessel revascularization					
Any	79 (10.5)	63 (8.4)	1.27 (0.91–1.77)	1.49 (1.05–2.10)	0.025
Related to MI	20 (2.7)	10 (1.3)	2.01 (0.94–4.29)	2.46 (1.13–5.38)	0.024
Not related to MI	63 (8.4)	57 (7.6)	1.11 (0.78–1.59)	1.31 (0.90–1.90)	0.17
Death					
All-cause	24 (3.4)	32 (4.3)	0.76 (0.45–1.28)	0.77 (0.45–1.33)	0.34
Cardiac cause	12 (1.6)	21 (2.8)	0.57 (0.28–1.16)	0.62 (0.30–1.27)	0.19
Myocardial infarction					
Non-fatal	24 (3.2)	19 (2.6)	1.26 (0.69–2.31)	1.56 (0.83–2.91)	0.17
Stent thrombosis					
Definite	5 (0.7)	6 (0.8)	0.83 (0.25–2.71)	1.33 (0.39–4.60)	0.65
Definite/probable	6 (0.8)	8 (1.1)	0.74 (0.26–2.15)	1.17 (0.39–3.50)	0.79
Composite endpoints					
Cardiac death, non-fatal MI, or TVR not related to MI	95 (12.5)	96 (12.6)	1.00 (0.75–1.33)	1.17 (0.87–1.57)	0.31
Cardiac death, non-fatal MI, or definite/probable ST	36 (4.8)	36 (4.7)	1.00 (0.63–1.58)	1.15 (0.71–1.85)	0.57

*CI* confidence interval, *MI* myocardial infarction, *SCC-BMS* silicon carbide-coated bare-metal stent, *ST* stent thrombosis, *UC-BMS* uncoated cobalt chromium bare-metal stent

^a^Adjusted for baseline and procedural characteristics using inverse probability weighting

### Sensitivity analysis

**Fig. 2:**
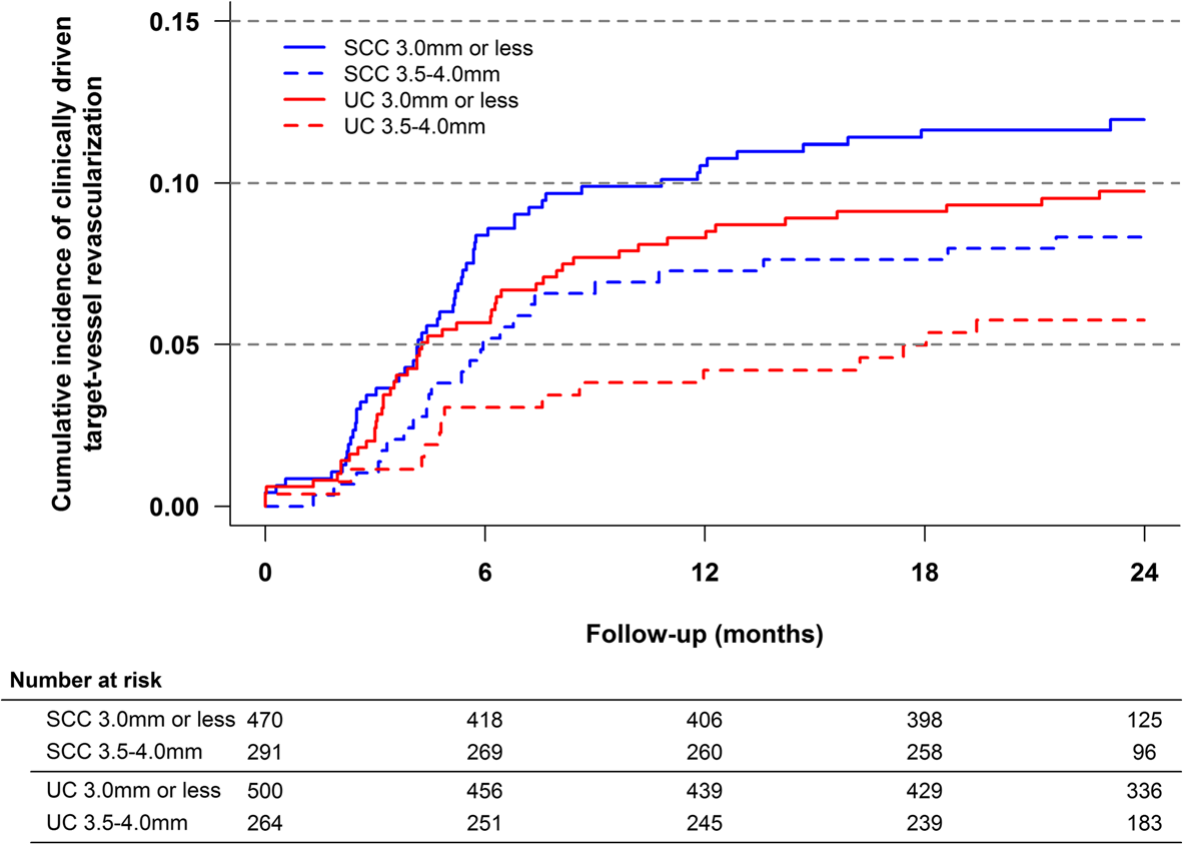
Cumulative incidence for clinically driven target-vessel revascularization within 24 months, by stent size and treatment group. In the SCC-group a stent size of ≤3.0 mm equaled a strut thickness of 60 μm, whereas a stent size of 3.5–4.0 mm conferred with a strut thickness of 80 μm. In the UC-group strut thickness was fixed at 81 μm regardless of stent size

The previously mentioned relation between stent diameter and strut thickness enabled an analysis of the influence of strut thickness on the risk of clinically driven TVR. Each treatment group was subdivided into patients receiving at least one stent ≤3.0 mm (SCC 60 μm vs. UC 81 μm) or only receiving stents 3.5–4.0 mm (SCC 80 μm vs. UC 81 μm). As shown in Fig. 2, the cumulative incidence curves for clinically driven TVR diverged between 5 and 8 months regardless of stent diameter and thus strut thickness. The stratified analyses yielded comparable size and direction of effect estimates: stent diameter ≤ 3.0 mm (cumulative incidence, 11.9% vs. 9.7%; adjusted HR, 1.43 [CI 95%, 0.97–2.11]; *p* = 0.071) and stent diameter 3.5–4.0 mm (cumulative incidence, 8.3% vs. 5.8%; adjusted HR, 1.72 [CI 95%, 0.85–3.47]; *p* = 0.13). Thus, the pooled estimate of the stratified analysis was similar to that of our main analysis (adjusted HR, 1.48 [95% CI, 1.06–2.06]; *p* = 0.022).

## Discussion

### Key findings

The main findings of this sub analysis from the BASKET-PROVE and BASKET-PROVE II trials with similar design, inclusion- and exclusion criteria, and endpoint definitions were as follows. In patients undergoing stenting of large coronary vessels, receipt of a SCC-BMS was associated with a higher risk of target-vessel revascularization compared to a UC-BMS. This difference was mainly driven by target-vessel revascularizations related to myocardial infarction. Differences in strut thickness did not explain these findings. No significant differences in ischaemic cardiac events were observed.

### Patient and procedural characteristics

Observed discrepancies in patient- and procedure-related characteristics were quite surprising provided that in- and exclusion criteria in the BASKET-PROVE trials were almost identical. Notably, the overall complexity of procedures in the SCC-BMS group was lower despite a higher prevalence of several cardiac risk factors compared to the UC-BMS group. Two important aspects of the BASKET-PROVE trials should be noted: [1] only six of the 11 study sites from the indigenous BASKET-PROVE trial went on to participate in the BASKET-PROVE II trial, and [2] the two trials were conducted 3 years apart. These factors may have changed the composition of potentially eligible patients in terms of patient- and procedure-related characteristics.

### Efficacy

No randomized comparison of the PRO-Kinetic and the Vision stents exist. Recent results of the BIOHELIX-I prospective study demonstrated 9-month rates of ischaemia-driven TVR of 7.26% in patients with stable or unstable CAD [18]. As patients with myocardial infarction were excluded, these results are not directly relatable to our study. The Canadian PRO-Vision study and the German COBALT registry both reported 3.1–3.8 percentage points higher rates of TVR at 12 and 18 months when using the PRO-Kinetic versus the Vision stent [7, 8]. We found a 2.1 percentage point higher risk of TVR at 2 years conferring with an adjusted relative risk of 49%. Direct comparisons of the results are hampered by differences in baseline and procedural characteristics including smaller vessels in the aforementioned studies which may at least in part explain the lower rates of TVR observed in our study at 24 months.

Interestingly, the higher rate of clinically driven TVR in the SCC group was at least in part driven by MI-related TVR. Numerically more patients in the SCC group experienced a non-fatal MI during follow-up; in this subgroup more than 80% of patients in the SCC group underwent MI-related TVR as compared to just above 50% in the UC group. Whether this observation represents a shift in practice over time, lesion characteristics or better handling capabilities of the PRO-Kinetic stent cannot be further elucidated using our data. A closer look at the cumulative incidence curves for MI-related and non-MI related TVR confirms that both curves diverge between 5 and 8 months. This observation is consistent with findings from the COBALT registry which demonstrated a dissociation of the cumulative incidence curves at 6 to 9 months [7].

### Pathophysiological considerations

Interactions between stent and vessel wall are highly complex. In context of findings from the COBALT registry our data suggest uninhibited neointimal hyperplasia as the main underlying pathophysiological mechanism [19]. Several factors may facilitate this process including strut thickness, stent design and strut coating, all of which merit further discussion. Strut thickness has been related to burden of neointimal atherosclerotic changes [20] and restenosis rates independent of stent design [21, 22]. Our sensitivity analysis did not provide any solid evidence that differences in strut thickness confounded or modified the relation between type of BMS and clinically driven TVR. To the best of our knowledge no direct comparisons between the double-helical and multilink architecture have been performed. In a rabbit model, Rogers et al. observed that changing the stent configuration by reducing strut-strut interconnections by 29% while holding diameter, mass, surface area, and stent surface material constant reduced vascular injury, thrombosis and neointimal hyperplasia significantly [23]. Changing surface material while holding mass, configuration, and diameter constant eliminated thrombosis, but left vascular injury and neointimal hyperplasia unchanged. These observations were supported by clinical findings emphasizing that stent design plays an important role in reducing the risk of in-stent restenosis and thrombus formation [24]. The silicon-carbide coating has been shown in vitro and in animal studies to reduce thrombogenicity and accelerate endothealization [4]. Theoretically, the silicon-carbide acts as a passive coating limiting the diffusion of metallic ions such as nickel and preventing interactions between the metallic body of the stent and cell surfaces, including thrombocytes and leucocytes. Our data did not allow us to discern the effect of stent design and the passive coating, but observations from the BIOFLOW V trial may provide clues to this problem [25]. The trial established non-inferiority in terms of target-lesion failure and demonstrated lower rates of target-vessel myocardial infarction in the Orsiro bioresorbable polymer sirolimus-eluting stent (PRO-Kinetic DES counterpart) versus the Xience durable polymer everolimus-eluting stent (Vision DES counterpart). Overall, these observations indicate that the SCC passive coating rather than stent design may have played a key role in our findings; a statement supported by the lack of clinical data on the effects of the silicon-carbide passive coating from human subjects.

### Safety

Rates of ischaemic cardiac events were consistent with those reported in prior studies [6, 7]. We did not find any significant differences in cardiac death, non-fatal MI or stent thrombosis between the two stent groups but may very well have lacked the power to do so. Importantly, the design of our study with different combinations of dual antiplatelet therapy (prasugrel- vs. clopidogrel-based) made it impossible to disentangle the effects of stent type and P_2_Y_12_-inhibitors. The TRITON-TIMI 38 trial demonstrated reduced rates of ischaemic events in patients with ACS undergoing PCI when using prasugrel versus clopidogrel as antithrombotic therapy [26]. Overall, our data on safety should be interpreted with caution.

### Study limitations

This post-hoc analysis should be interpreted in the context of the certain limitations and considered hypothesis-generating. Target-lesion revascularization (TLR) was not measured in the BASKET-PROVE trials. However, TVR and TLR have been shown to correlate well [27]. Routine angiography was not performed during follow-up as per protocol. Although propensity score based methods have been shown to remove more than 90% of the overt bias due to the covariates used to estimate the score when used properly, it cannot remove any hidden bias [16]. Given the high quality and compatibility of our data from the BASKET-PROVE trials we deem that any major influence of unmeasured confounding was unlikely. The BASKET-PROVE trials were conducted 3 years apart meaning that we cannot rule out bias induced by potential changes in practice; however, the fact that the two study protocols were almost identical and 77% of patients were enrolled at study sites participating in both trials limits the magnitude of such bias. Finally, the BASKET-PROVE trials do not necessarily reflect contemporary practice as the latest trial ended 7 years ago.

## Conclusion

In patients requiring stenting of large coronary vessels ≥3.0 mm, the use of SCC-BMS was associated with an increased risk of clinically driven target-vessel revascularization at 2 years compared to UC-BMS; primarily due to repeat revascularizations related to myocardial infarction. Irrespective of the underlying causative mechanism, stent coating or stent design, these findings emphasize the need for rigorous testing before adopting new devices into clinical practice. Specifically, follow-up well beyond 6 months seems advisable when assessing safety and efficacy of new stents.

## Supplementary information

**Additional file 1: Figure S1**. Covariate balance before (unadjusted) and after (adjusted) inverse probability weighting of the study population. *A Love plot displaying covariate balance between the treatment groups prior to and after applying inverse probability weighting.* **Figure S2**. Cumulative incidence curves for clinically driven target-vessel revascularization related to myocardial infarction and not related to myocardial infarction. *Displays cumulative incidence curves for individual components of the primary endpoint, by BMS-group.* **Figure S3**: Cumulative incidence curves for cardiac death, non-fatal myocardial infarction and definite/probable stent thrombosis. *Displays cumulative incidence curves for individual components of the main secondary endpoint, by BMS-group*

## Data Availability

The data that support the findings of this study are available from the BASKET-PROVE and BASKET-PROVE II steering committees, but restrictions apply to the availability of these data, and so are not publicly available. Data are however available from the authors upon reasonable request and with permission of the BASKET-PROVE and BASKET-PROVE II steering committees.

## Abbreviations

BMS: Bare-metal stent
CABG: Coronary artery bypass-grafting surgery
CAD: Coronary artery disease
DAPT: Dual antiplatelet therapy
MI: Myocardial infarction
NSTE-ACS: Non-ST-segment elevation acute coronary syndrome
PCI: Percutaneous coronary intervention
ST: Stent thrombosis
TVR: Target-vessel revascularization
