# Supplementary material for: Do ultrathin strut bare-metal stents with passive coating improve efficacy in large coronary arteries? Insights from the randomized, multicenter BASKET-PROVE trials
Source: BMC Cardiovasc Disord. 2019 Oct 16;19:226. doi: 10.1186/s12872-019-1199-8 (PMC6796469; doi:10.1186/s12872-019-1199-8)

**Supplementary Appendix**

**Do ultrathin strut bare-metal stents with passive coating improve efficacy in large coronary arteries?**

**Insights from the randomized, multicenter BASKET-PROVE trials**

Kim Wadt Hansen, MD PhD; Raban Jeger, MD; Rikke Sørensen, MD PhD; Christoph Kaiser, DMSci.; Matthias Pfisterer, DMSCi.; Tor Biering-Sørensen, MD PhD; Louise Bjerking Hougesen, MD; Søren Galatius, DMSci.

**Appendix Figure 1 Covariate balance before (unadjusted) and after (adjusted) inverse probability weighting of the study population**

**
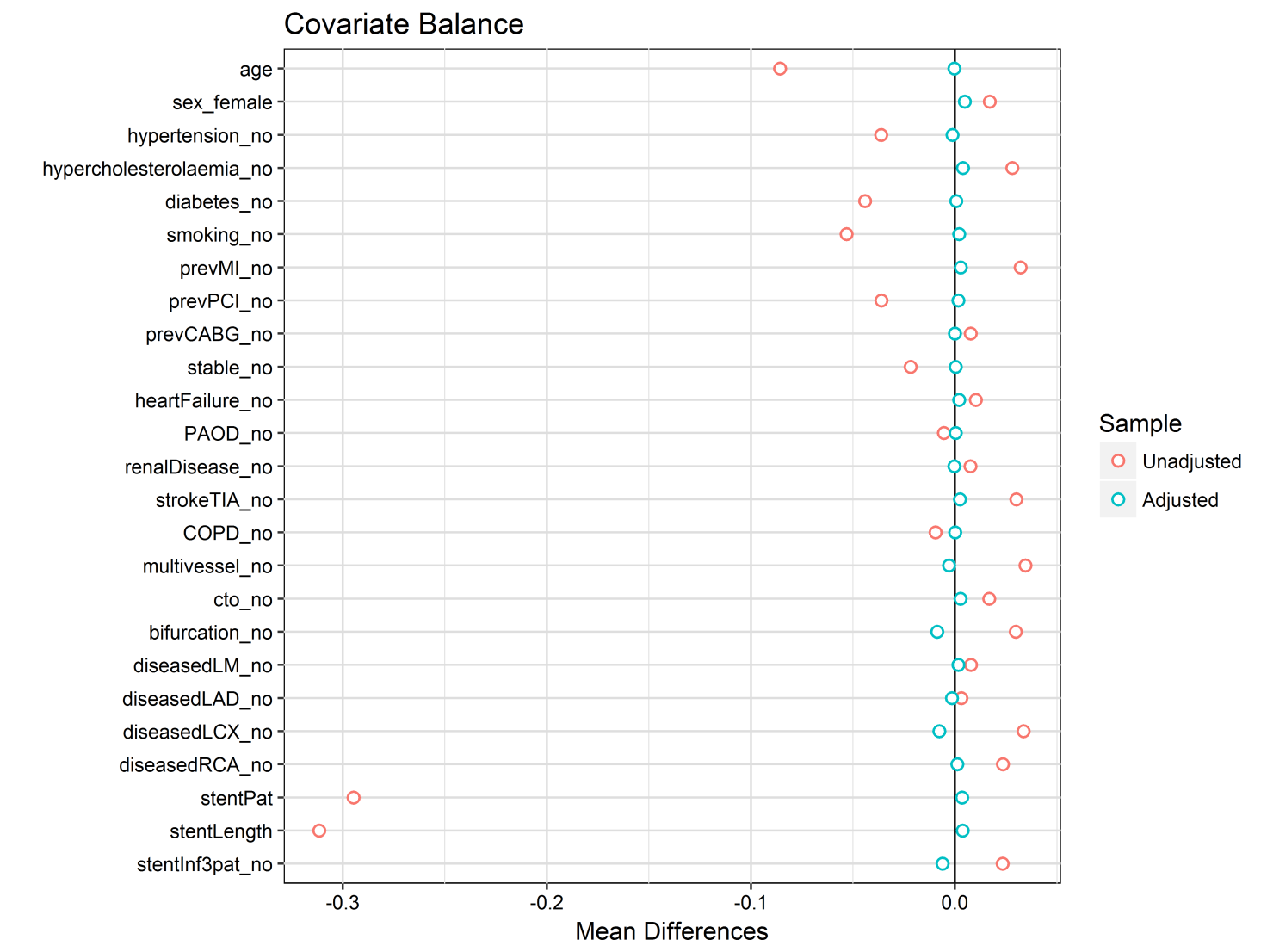
**

**Appendix Figure 2 Cumulative incidence curves for clinically driven target-vessel revascularization related to myocardial infarction and not related to myocardial infarction**


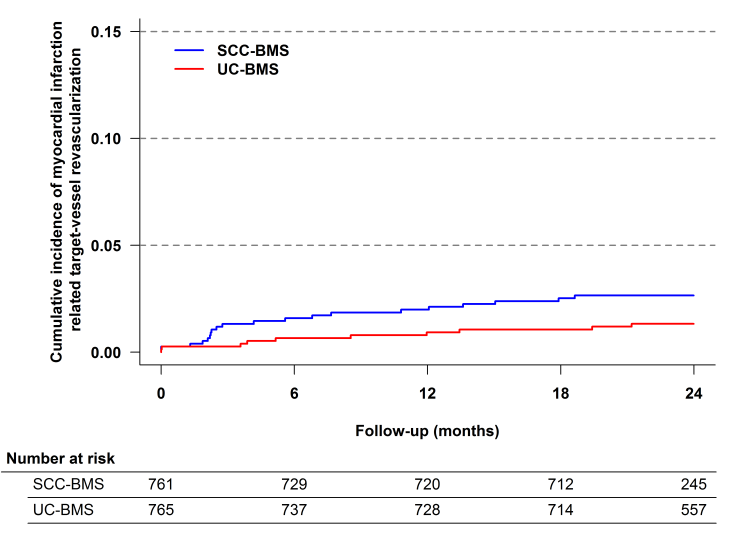

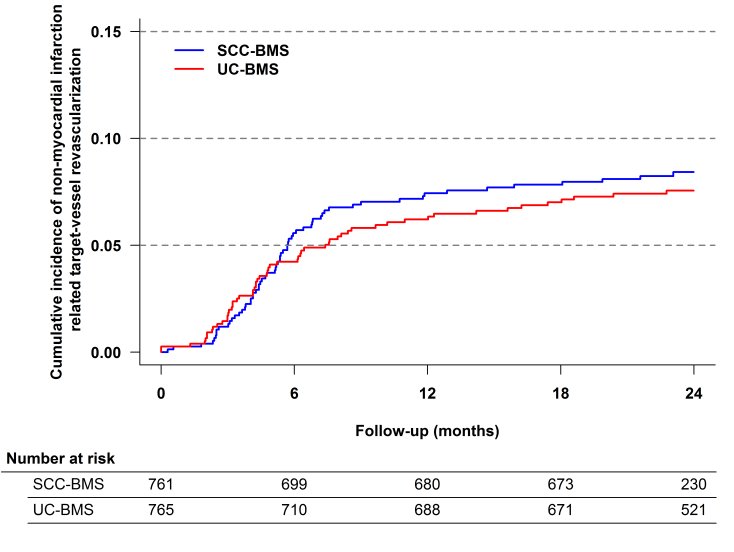


**Appendix Figure 3 Cumulative incidence curves for cardiac death, non-fatal myocardial infarction and definite/probable stent thrombosis**


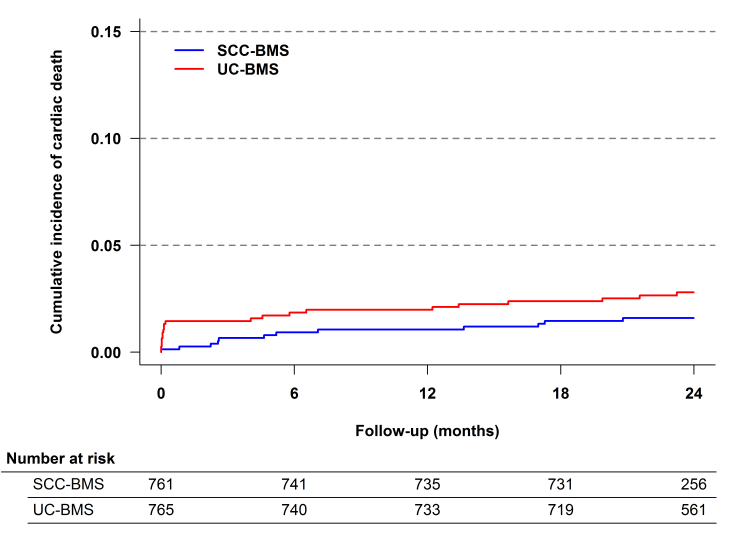

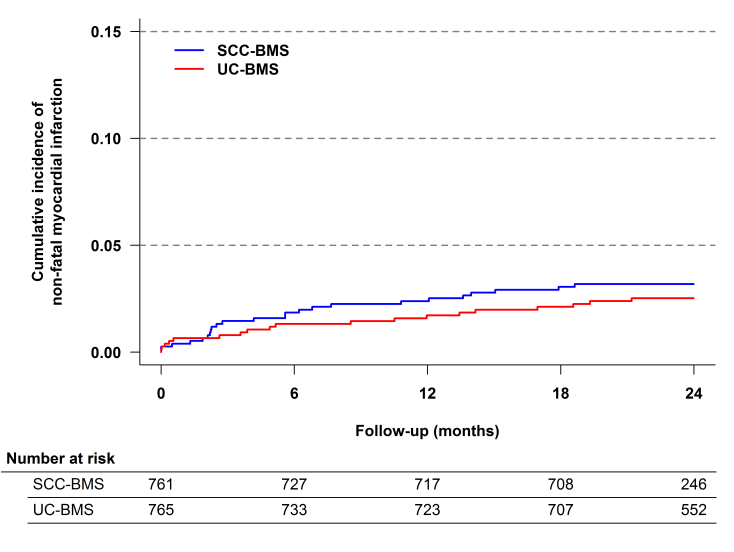

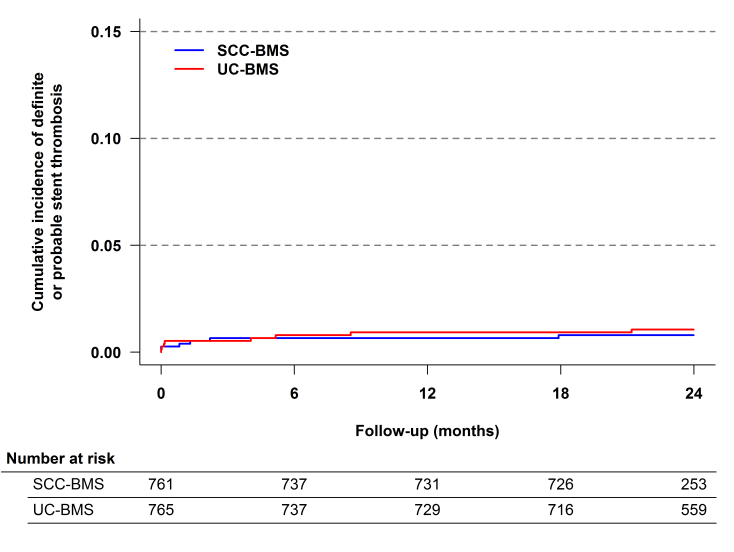

Supplement: Supplementary file 1 — Additional file 1: Figure S1. Covariate balance before (unadjusted) and after (adjusted) inverse probability weighting of the study population. A Love plot displaying covariate balance between the treatment groups prior to and after applying inverse probability weighting. Figure S2. Cumulative incidence curves for clinically driven target-vessel revascularization related to myocardial infarction and not related to myocardial infarction. Displays cumulative incidence curves for individual components of the primary endpoint, by BMS-group. Figure S3: Cumulative incidence curves for cardiac death, non-fatal myocardial infarction and definite/probable stent thrombosis. Displays cumulative incidence curves for individual components of the main secondary endpoint, by BMS-group [file 12872_2019_1199_MOESM1_ESM.docx]
